# Beyond survival after pulmonary metastasectomy: functional preservation, patient-reported outcomes, and the cumulative burden of repeat resection

**DOI:** 10.3389/fmed.2026.2003447

**Published:** 2026-10-26

**Authors:** Zhe Li, Baolei Lyu

**Affiliations:** Department of Thoracic Surgery, Shijiazhuang People’s Hospital, Shijiazhuang, Hebei, China

**Keywords:** future local-treatment eligibility, oligometastatic disease, parenchyma-sparing surgery, patient-reported outcomes, pulmonary function, pulmonary metastasectomy, quality of life, repeat metastasectomy

## Abstract

Pulmonary metastasectomy (PM) is used in selected patients with pulmonary metastatic disease, but survival-focused reporting incompletely describes recovery and cumulative treatment burden. Following the parenchymal-preservation and repeatability principles discussed by Scanagatta et al. in 2024, this narrative review examines pulmonary function, patient-reported outcomes, repeat resection, treatment sequencing, and retained local-treatment options. A purposive synthesis was updated with six focused PubMed searches on 8 October 2026, including recent surgical studies and function and quality-of-life evidence for non-surgical local treatments. Functional cohorts document early impairment and variable recovery after PM; pulmonary-function data after stereotactic radiotherapy also caution against assuming that avoidance of resection means avoidance of functional loss. Contemporary repeat-resection and modality-comparison studies remain predominantly retrospective and do not establish equivalent recovery or causal survival benefit. Colorectal, sarcoma, and mixed-primary evidence is interpreted separately. We propose “future local-treatment eligibility” as a modality-specific record of technical feasibility, physiological tolerability, and oncological appropriateness, accompanied by a separate assessment of informed patient preference. “Future operability” denotes the surgical component. This organizing construct is unvalidated. Treatment-related limitations must be distinguished from progression, competing illness, patient choice, and access barriers; non-receipt of treatment cannot establish loss of capacity. Candidate longitudinal outcomes and illustrative assessment windows are proposed for stakeholder consensus and feasibility testing, not as an established core outcome set. Histology-specific prospective cohorts with serial function and symptom measurements, inclusive recurrence denominators, and explicit reasons for treatment decisions are needed to evaluate what patients retain after initial and repeat PM.

## 1 Introduction

Pulmonary metastasectomy (PM) is used within multidisciplinary care for selected patients with pulmonary metastatic disease. Contemporary practice emphasizes complete resection with preservation of healthy parenchyma and the possibility of retreatment (1). The International Registry of Lung Metastases documented long-term survival in selected surgical patients (2), while subsequent expert consensus supported individualized consideration and acknowledged the need for stronger comparative evidence (3). Survival after an operation, however, does not identify the survival attributable to that operation.

The PulMiCC randomized trial illustrates this uncertainty in colorectal cancer (CRC). Recruitment limitations prevented a precise estimate of treatment effect (4). In the updated analysis of 93 randomized patients, median survival was 3.5 years after metastasectomy and 3.8 years with active monitoring (5). The wide uncertainty does not establish equivalence or absence of benefit. It does challenge the assumption that selected patients necessarily have negligible survival without PM. Local treatment should consequently be assessed within a disease course that may also involve systemic therapy, surveillance, stereotactic body radiotherapy (SBRT), thermal ablation, and further interventions.

Scanagatta et al. emphasized precision resection, parenchymal preservation, and repeatability in their 2024 clinical review (1). These are established surgical principles rather than concepts introduced here. Our proposed contribution is to organize their evaluation longitudinally: measure recovery, cumulative burden, and the reasons a specific treatment remains available or becomes unsuitable at recurrence. Preservation of tissue is an intended mechanism; preserved function and retained treatment eligibility are outcomes that require observation.

In PulMiCC, serial EQ-5D-3L measurements did not detect a health-utility advantage for surgery (6); this is not evidence of equivalent quality of life. Its burden-of-care analysis also showed baseline differences between treatment groups and no clear reduction in chemotherapy use with repeated local intervention (7). These findings support measuring recovery and treatment exposure directly rather than inferring them from survival or procedural success.

Functional consequences reflect resected parenchyma, surgical access, and the wider treatment and disease context (8). These influences interact: an open approach may permit palpation-guided limited resections, whereas multiple deep wedges can consume substantial lung despite minimally invasive access. Systemic therapy, pre-existing lung disease, recurrence, and deconditioning further modify recovery. Baseline measurements and assessment timing are therefore essential.

Prospective PM studies have documented postoperative pain, dyspnea, fatigue, and impairment of physical and role function, with different recovery patterns over three to twelve months (9, 10). A dedicated 2025 review by Duarte D’Ambrosio et al. has already examined patient-reported outcome measures in PM (11). The present review complements that work by connecting symptoms and physiological recovery to repeated-treatment exposure and decisions at recurrence; patient-centered assessment itself is not presented as novel.

We use future local-treatment eligibility as a proposed record of modality-specific technical feasibility, physiological tolerability, and oncological appropriateness, accompanied by a separate assessment of informed patient preference. Future operability is its surgical component, not a synonym for eligibility for SBRT or ablation. Preference affects the treatment decision but should not be confused with physiological or technical capacity. The construct is unvalidated; its four decision components should be recorded separately rather than combined into a score or an unexplained binary outcome.

The objectives are to synthesize functional and patient-reported evidence after initial and repeat PM, compare observed outcomes with the expected mechanisms of different local treatments, and propose candidate measurements for future studies. We distinguish findings in CRC, sarcoma, and mixed-primary cohorts. The measurement principles may be shared across histologies, but indications, expected benefit, and treatment sequencing cannot be assumed to be interchangeable.

## 2 Methods

### 2.1 Review design and scope

This is a narrative review of adult and relevant mixed-age PM cohorts, organized around the patient trajectory and treatment decisions. It is not a systematic or scoping review. Heterogeneity in histology, technique, follow-up, and outcome measurement precluded a pooled estimate. Table 1 identifies five empirical outcome domains and one proposed organizing domain.

**Table 1:** Cross-cutting outcome domains.

Domain	Examples of measurements	Interpretive purpose
Perioperative recovery	Complications; 30-/90-day mortality and readmission; activity recovery	Describe immediate burden and recovery rather than infer it from discharge.
Pulmonary function	FEV1, FVC, DLCO; oxygen use; indicated exercise testing	Quantify physiological trajectory with explicit units and baseline.
Patient-reported health	Dyspnea, pain, fatigue; physical and role function; global health	Describe the patient's experience and meaningful change.
Treatment sequencing	Systemic-treatment interruptions and resumption; subsequent local treatments	Link treatment episodes to the wider cancer pathway.
Healthcare use	Hospital days; outpatient and emergency care; rehabilitation; patient costs	Measure cumulative resource and time burden.
Future local-treatment eligibility — proposed	Modality-specific technical, physiological and oncological assessments; separate patient preference; reasons for limitations	Organize subsequent decisions; not an established empirical endpoint or validated score.

FEV1, forced expiratory volume in 1 s; FVC, forced vital capacity; DLCO, diffusing capacity for carbon monoxide; PRO, patient-reported outcome. The proposed eligibility domain is an organizing device, not an established extraction instrument.

### 2.2 Information sources and search strategy

The original manuscript stated a PubMed/MEDLINE search cutoff of 18 September 2026. For the revision, searches were updated and broadened through NCBI E-utilities on 8 October 2026. Six focused queries combined pulmonary or lung metastases/metastasectomy with terms for function and patient-reported health, repeat resection, surgery versus stereotactic body or ablative radiotherapy (SBRT/SABR), and thermal ablation. Three queries emphasized publications from 2023 onward; complementary queries covered earlier surgical functional studies and non-surgical functional/PRO evidence. Retrieval yielded 234, 63, 89, 127, 120, and 95 records, respectively, comprising 617 unique PubMed identifiers. Supplementary Material S1 provides every executed search string, date range, selection priority, and source-access level; the accompanying log retains PubMed translations and identifiers. These are retrieval counts, not a systematic-review inclusion flow.

### 2.3 Eligibility and prioritization

Selection was purposive and not independently duplicated. We prioritized direct PM functional or patient-reported measurements, contemporary repeat-resection cohorts, and direct surgery–SBRT comparisons. Pulmonary-metastasis SBRT and ablation studies were examined to test modality-specific assumptions about recovery and subsequent options. Prospective measurements, randomized companion reports, and informative contemporary cohorts received particular attention. Bibliographic follow-up and targeted publisher/repository searches supplemented PubMed. Reviews supplied context rather than additional independent patient samples. Pediatric-only series, isolated case reports, and reports without a direct contribution to the clinical questions were not prioritized. Mixed-organ oligometastatic studies were used only as explicitly indirect context. English-language reports were used. Full texts were consulted when accessible; otherwise, extraction was confined to the published abstract, as identified in S1.

### 2.4 Data mapping

Study design, sample size and unit, primary histology, intervention, outcome instrument, assessment timing, findings, and limitations were mapped to the outcome domains. Table 2 summarizes selected primary studies rather than an exhaustive inventory. Companion publications and potentially overlapping cohorts were not treated as independent replications. No patient totals or pooled effects were calculated. Future local-treatment eligibility was used to identify missing decision information, not as an established endpoint presumed to have been measured. The treatment-based sections examine these cross-cutting domains where the evidence is most directly applicable.

**Table 2:** Selected primary evidence for longitudinal outcomes.

Study and design	Sample and histology	Intervention	Outcomes and follow-up	Principal finding	Main limitation
Petrella 2009 (14) Retrospective	66 patients; mixed primaries	First PM, with reassessment before a further operation	Spirometry and transfer coefficient; variable interval, mean 54.5 days	Impairment related to resection burden and interval between assessments	Selected for another operation; not a standardized recovery cohort
Welter 2012 (15) Prospective observational	162 enrolled; 117 analyzed; mixed primaries	Wedge, segmental or lobar PM; 33 bilateral cases	Spirometry; DLCO in 103; mean follow-up 3.4 months	Mean FEV1 87.0 to 76.2% predicted, −10.8 percentage points	45 excluded for several reasons; older era; potential overlap with other Welter reports
Welter 2016 (16) Post-hoc cohort analysis	31 bilateral cases; mixed primaries	Staged bilateral PM	Pulmonary-function tests at baseline and 3 months; early tests in 15	Persistent impairment at 3 months; early loss greatest after second stage	Small subgroup; staged treatment differs from metachronous repeat PM
Hassan 2021 (17) Prospective bicentric	61 patients; 77 operations; mixed primaries	Laser-assisted PM, predominantly thoracotomy	Spirometry and DLCO; 3 and 6 months	Reduced function at 3 months; limited further recovery overall	44 and 38 patients tested; metric-specific missingness and no comparator
Baum 2024 (18) Retrospective database study	126 patients; mixed primaries	Unilateral laser-assisted PM	Serial FEV1 through 12 months	Median FEV1 98% predicted at baseline and 96% at 12 months	Additional lung surgery excluded; non-significance does not establish complete recovery
Welter 2013 (9) Prospective	126 cases; mixed primaries	Open PM	EORTC QLQ-C30/LC13; baseline and 3 months	Physical/role function and several symptoms deteriorated	No randomized access comparison; limited follow-up window
Balduyck 2012 (10) Prospective comparative	35 patients; mixed primaries	Standard PM (n = 27); isolated lung perfusion plus PM (n = 8)	EORTC instruments; 1, 3, 6, 12 months	Standard-PM group reported domain recovery by 6 months	Very small non-randomized groups; not proof of individual recovery
Brew-Graves 2021 (6) Randomized companion report	93 randomized; 91 returned questionnaires; CRC	PM versus active monitoring	EQ-5D-3L; baseline, 3, 6, 12, 24 months	No statistically detected utility advantage	Imprecision and longitudinal missingness; not an equivalence trial
van Dorp 2023 (35) National registry cohort	1,237 patients; 127 repeat PM; CRC	First versus repeat PM	Complications, survival; median follow-up 42 months	Complications 18.1% after repeat versus 11.6% after first PM	Selected repeat group; no serial PRO/function comparison
Mills 2023 (36) Retrospective	117 patients reaching at least a third PM; non-lung primaries	Third and further operations	Blood loss, complications, stay, readmission per procedure	Greater third-procedure blood loss and wound complications	Strong survivor selection; no serial functional endpoint
Ferrari 2026 (37) Retrospective single center	1,106 patients; 1,503 resections; CRC, sarcoma and others	Single, multiple and repeated PM	Survival and hospital stay; median 9.5 years among survivors	Long-term outcomes reported in selected repeat-treatment patients	Treatment era 2003–2018; no serial PROs or untreated counterfactual
Kakibuchi 2026 (43) Retrospective	60 sarcoma patients; 31 second pulmonary recurrences; 16 repeat PM	Initial and repeat PM	Survival and recurrence interval; 5-year OS reported	Disease-free interval associated with selection and prognosis	Small recurrence subgroup; data-derived cutoff not validated
van Dorp 2023 (54) Retrospective two center	100 CRC patients; 159 lesions	Surgery 40 patients; SABR 60	Survival/local failure; median 38 versus 46 months	Lesion recurrence 7.2% after surgery versus 31.1% after SABR	Center preference, dose and selection; no comparative serial PROs
Lee 2025 (56) Retrospective single center	404 CRC patients; 528 lesions	Wedge resection 334; SABR 70	Local control/toxicity; median 40.8 versus 23.7 months	3-year local control 88.6% versus 86.7%; severe pneumonitis in 2 SABR patients	Imbalanced disease burden and follow-up; not an equivalence study
Song 2025 (online 2024) (57) Retrospective comparative	75 patients in first-line survival analysis; sarcoma	Surgery 61; SABR 14	Local control/survival; median 44.8 months for survival analysis	No statistically detected differences in selected outcomes	Median age 54 versus 73 years; small SABR group; no functional comparison
van Dorp 2023 (50) Retrospective salvage series	17 CRC patients; 20 operations	Salvage PM after SABR failure	Surgical morbidity, extent and survival after salvage	Grade III–IV morbidity after 4/20 operations; 90-day mortality zero	Only patients reaching salvage included; source abstract did not specify follow-up duration
ECLIPSE 2015/2021 (60, 61) Prospective single arm	40 patients; 60 lesions; mixed primaries	Percutaneous cryoablation	Local control, SF-12 and performance status; through 5 years	Local control and longitudinal health outcomes were measurable	Small selected cohort, attrition, no surgery comparator; companion reports are one cohort
Bucknell 2024, SAFRON II (48) Randomized fractionation trial analysis	90 randomized; 87 treated; 133 pulmonary oligometastases	SBRT 28 Gy in 1 fraction versus 48 Gy in 4	Modeled serial PFTs; 12-month changes reported	FEV1 −0.17 L and DLCO −1.7 mL/min/mmHg at 12 months	No surgery or untreated arm; target multiplicity and intervening disease matter
Aksoy 2026 (49) Retrospective	55 patients; 71 lesions; mixed primaries including NSCLC	SBRT	PFTs at baseline and 3 months	13/55 met the study-defined FEV1 decline threshold	Short follow-up; no comparator; threshold not validated for PM; abstract extraction
Callstrom 2020, SOLSTICE (62) Prospective multicenter single arm	128 patients; 224 lung metastases; 169 procedures	Percutaneous cryoablation	SF-12 at 1 and 3 months; tumor follow-up through 24 months	Pleural catheter after 44/169 procedures; no significant mean SF-12 general-health change	Selected small lesions; no surgery comparator or validated later-eligibility outcome
Kim 2026 (51) Retrospective single center	17 selected cases; 12 CRC, 2 primary lung cancer, 3 other	Salvage resection after SABR local failure	Operative findings and morbidity; median follow-up 58.1 months	Two conversions to thoracotomy, including one for severe adhesions	Mixed primary/secondary malignancy; operated patients only; no causal attribution

Selected primary studies relevant to the review questions, not an exhaustive catalogue. Sample sizes distinguish patients, lesions, and procedures. Follow-up refers to the reported outcome window or cohort median, not uniform observation for every patient. CRC, colorectal cancer; PM, pulmonary metastasectomy; SABR, stereotactic ablative radiotherapy; PRO, patient-reported outcome; OS, overall survival. Non-significant findings do not establish equivalence or complete recovery. Full-text versus abstract access and limitations are detailed in S1. Some older cohorts may overlap; no totals or pooled estimates are calculated. PFT, pulmonary-function test; NSCLC, non-small cell lung cancer; SF-12, 12-Item Short Form Health Survey. The Welter reports arise from related clinical settings and may overlap; SABR-5 and OligoCare are discussed separately as mixed-organ context. Publication year is the issue year, with an earlier online year specified when relevant.

### 2.5 Evidence synthesis and appraisal

Interpretive weight depended on directness to PM, prospective measurement, baseline adjustment, validated instruments, follow-up completeness, treatment selection, and the handling of repeated observations. No formal risk-of-bias scale or certainty-grading system was applied. Study-specific limitations are displayed in Table 2. Observational survival associations were treated as prognostic rather than causal. Failure to measure harm is not evidence of preserved function, and non-significance does not establish recovery, equivalence, or non-inferiority.

### 2.6 Terminology and analytic framework

Parenchyma-sparing and precision resection describe lesion-directed strategies intended to achieve oncologically adequate clearance while avoiding unnecessary loss of functioning lung. Neither implies a particular device, margin threshold, or access route. Repeat PM denotes a subsequent resection for recurrent or new pulmonary metastases, distinct from planned staged bilateral surgery where the source permits this distinction. Cumulative burden includes repeated tissue loss, access-related morbidity, complications, recovery time, and disruption of other treatment. Eligibility for a later treatment is separated from whether that treatment was offered, chosen, scheduled, or delivered.

The proposed trajectory is baseline assessment, index treatment, recovery, recurrence or progression, and reassessment. At each decision point, reasons for limitations are classified as treatment-related, disease-related, patient-related, or access-related, with multiple and uncertain causes permitted. Death is recorded separately. No recurrence or no present indication for intervention does not constitute treatment ineligibility.

### 2.7 Limitations of the review

The targeted update improves traceability but cannot establish literature completeness. Single-database searching, purposive selection, English-language restriction, incomplete full-text access, and absence of duplicate screening or formal certainty grading introduce selection and reporting risks. Older cohorts may not reflect contemporary systemic treatment, imaging, or minimally invasive practice. Histological and methodological heterogeneity, informative loss to follow-up, and under-reporting of non-treated patients limit generalization. The eligibility construct and candidate outcome framework are proposals without stakeholder consensus, feasibility testing, or validation; they should not be used to rank treatments or determine care independently of established clinical assessment.

## 3 Why conventional outcomes are insufficient

### 3.1 Survival after surgery is not the same as survival caused by surgery

Overall survival and disease-free survival remain essential, but observational PM series do not supply the untreated counterfactual (4, 5, 12). For example, a 15-center European cohort of 1,647 patients reported R0 resection in 95.7% and 30-day mortality of 0.4% (13). These results describe procedural outcomes in selected patients. They do not quantify the effect of surgery on survival, symptoms, or retained treatment capacity.

### 3.2 Perioperative safety does not describe the longitudinal treatment burden

Perioperative mortality, complications, and length of stay measure acute safety, not restoration of pre-treatment health. The prospective quality-of-life studies and randomized utility data show why symptom domains and measurement windows matter (6, 9, 10). Persistent symptoms can coexist with an uncomplicated recovery by conventional surgical definitions. Repeated procedures should therefore be described per episode and cumulatively per patient (7).

### 3.3 Denominators determine what can be inferred

Repeat-PM cohorts select patients who survive, recur in a treatable pattern, remain fit, and agree to another procedure. Studies should retain both the original population considered for treatment and, separately, all patients with pulmonary recurrence. At recurrence, the denominator must include patients receiving systemic therapy, observation, supportive care, or no treatment as well as those receiving another local intervention. Reasons for non-treatment are necessary; the proportion not reoperated cannot itself measure treatment-related loss of eligibility.

Functional and patient-reported follow-up also requires explicit denominators. Death, progression, further therapy, and illness-related non-response can make observed survivors appear healthier. Report how many patients were expected and assessed at each visit, reasons for missingness, and intervening treatment. Table 2 brings together the principal primary evidence relevant to these longitudinal questions.

## 4 Pulmonary function after metastasectomy

### 4.1 Early loss and recovery are both plausible

Available studies document functional loss, but their assessment windows differ. Petrella et al. studied 66 patients selected for another pulmonary operation; spirometry was repeated before that procedure at variable intervals averaging 54.5 days (14). This is not a standardized recovery curve in an unselected cohort. In a prospective cohort, 117 of 162 enrolled patients completed follow-up at a mean of 3.4 months (15). Mean forced expiratory volume in 1 s (FEV1) decreased from 87.0% to 76.2% predicted, a difference of −10.8 percentage points. Bilateral resection was associated with greater spirometric loss and postoperative chemotherapy with impaired gas transfer. The 45 exclusions included patients who did not undergo PM, loss to follow-up, illness, and deaths. A separate *post-hoc* report of 31 bilateral cases found impairment at three months (16). These reports may contain overlapping patients and are not independent replications or quantitatively comparable recovery trajectories.

In a prospective laser-assisted study of 61 patients and 77 operations, pulmonary-function measures were reduced at three months with little additional cohort-level improvement by six months; only 44 and 38 patients underwent testing at those respective visits (17). Baum et al. subsequently reported a different pattern in 126 selected unilateral laser-resection patients (18). Median FEV1 was 98% predicted before surgery, 71% near discharge, 90% at three months, 93% at six months, and 96% at twelve months. The difference between the preoperative and twelve-month medians is −2 percentage points of predicted FEV1, not a 2% relative loss and not a paired patient-level estimate. Non-significant late differences do not prove complete recovery. Patients undergoing additional lung surgery during follow-up were excluded, limiting application to repeat PM.

### 4.2 Extent should be measured, not inferred from the procedure label

These cohorts support measuring resection burden rather than inferring it from “wedge” or “laser.” Lesion count, depth, laterality, anatomical resection, previous operations, and actual or estimated tissue loss should be recorded (14–18). Imaging-derived volume measures may be useful research variables, but require reproducibility testing. The common physiological rationale for conserving lung does not establish an identical magnitude of benefit across histologies: lesion distribution, treatment exposure, and selection differ.

Pulmonary function reporting should distinguish absolute change in liters, percentage-point change in percentage-predicted values, and relative percentage change from baseline. For example, 80% to 72% predicted represents −8 percentage points and a −10% relative change: 100 × (72−80)/80. A difference between cohort medians is not the median of individual changes. Where published units are unclear, changes should remain qualitative rather than be silently converted. FEV1, forced vital capacity (FVC), and diffusing capacity for carbon monoxide (DLCO) measure different aspects of function; alveolar-volume-adjusted transfer coefficient is not interchangeable with DLCO.

### 4.3 Candidate measurement windows

For future feasibility work, we suggest a baseline assessment within four weeks before treatment, early recovery at approximately one month (a 2–6-week window), and follow-up at three and twelve months. This illustrative schedule is used consistently in Table 3 for both function and PROs; it is not a consensus recommendation. Restart the sequence for each new treatment episode, with event-triggered reassessment at recurrence or major treatment changes. Testing should be clinically appropriate, with timing deviations documented. Spirometry, DLCO, and field walking tests should use standardized methods (19–21).

**Table 3:** Candidate longitudinal outcomes and illustrative assessment windows.

Domain	Candidate variables	Illustrative timing	Reporting unit
Population and biology	Histology/subtype; disease tempo; systemic exposure; extrapulmonary control; lesion number, size and distribution	Initial MDT decision and each recurrence	Per patient and decision episode
Oncological outcomes	Margin for surgery; lesion-level control; pulmonary and systemic progression; survival	Capture event dates throughout follow-up; imaging according to the clinical protocol	Per lesion and per patient; report clustering
Safety and recovery	Complications; mortality/readmission; days alive and out of hospital; independent activity	Discharge; 30 and 90 days	Per episode and cumulative patient burden
Pulmonary function	FEV1, FVC, DLCO; oxygen use; indicated exercise test; units and individual change	Baseline within 4 weeks before treatment; about 1 month (2–6 weeks), 3 and 12 months	Per patient longitudinally; repeat for each episode
Patient-reported health	Pain, dyspnea, fatigue; global health; physical/role function; acceptability; work and caregiving	Same baseline, 1-month (2–6-week), 3- and 12-month windows	Per patient; instrument-specific meaningful-change criteria
Treatment and resource use	Regimens, interruptions/resumption, new lines; hospital/clinic days; rehabilitation; patient costs	Record transitions continuously; summarize at 30/90 days and 12 months	Per episode and cumulatively
Future local-treatment eligibility — proposed	For each modality: technical, physiological and oncological status; preference separately; reasons for limitations; offered/chosen/delivered treatment	At recurrence or major decision changes; reassess temporary limitations	Original decision cohort plus all recurrence cases; death reported separately

This is a candidate framework for stakeholder consensus and feasibility testing, not an established core outcome set or clinical schedule. The same functional/PRO windows are used in Sections 4.3 and 8.3; clinically necessary deviations should be recorded. Add event-triggered reassessment at recurrence or major treatment changes. A treatment episode means one index or repeat intervention; the patient-level analysis accumulates all episodes. Thirty- and ninety-day safety windows and continuous oncological follow-up answer different questions from functional recovery.

Report individual trajectories, confidence intervals, missing data, and clinically meaningful deterioration or recovery when validated interpretation criteria exist. A non-significant group mean cannot exclude persistent impairment in a subgroup. Symptoms and activity should be measured alongside physiology because pain, fatigue, and role limitation are not captured by spirometry alone (9, 10).

## 5 Surgical access and patient-reported recovery

### 5.1 Observed short-term outcomes and their limits

Thoracotomy enables manual palpation, while video-assisted thoracoscopic surgery (VATS) reduces the extent of access trauma. These procedural differences do not by themselves establish a durable patient-reported advantage. Patients with fewer, peripheral, unilateral lesions are often selected for minimally invasive surgery, whereas open access is used for more complex disease. Access and resection burden must therefore be considered together.

A 2024 meta-analysis of 11 observational studies and 2,159 patients associated VATS with a hospital stay approximately two days shorter than thoracotomy (22). Its survival association cannot be attributed to access alone. An earlier observational meta-analysis likewise did not establish a difference in recurrence-free survival (23). Neither result demonstrates equivalent oncological outcomes or superior long-term functional recovery. Such comparisons are especially sensitive to histology, lesion distribution, and the extent of resection.

### 5.2 Palpation, detection, and recovery represent a genuine trade-off

A randomized comparison of hand-assisted thoracoscopy and limited thoracotomy found less pain after withdrawal of epidural analgesia with the hand-assisted approach (24). Sequential and prospective palpation studies identified additional nodules after thoracoscopy or imaging, but additional nodules included benign lesions, and their removal has not been shown to confer a survival advantage (25–28). These studies address detection, not the net balance of recovery, additional resection, and cancer control.

Modern localization can support limited minimally invasive resections, as illustrated by a selected VATS cohort using high-resolution imaging and localization (29). It does not eliminate the need to measure missed lesions, repeat procedures, or symptoms. The relevant comparison is between complete treatment strategies for comparable patients, not simply incision types.

### 5.3 Patient-reported outcomes require direct measurement

In the prospective open-PM study of 126 cases, physical and role function, fatigue, pain, and dyspnea deteriorated at three months (9). In the smaller study of 35 patients, the 27 undergoing standard PM had reported recovery of measured quality-of-life domains by six months; the eight receiving isolated lung perfusion had a different symptom profile (10). These data should not be pooled into a universal expected recovery time. The 2025 PM-specific PROM review also makes clear that this is an existing field of investigation (11).

Comparative studies should collect baseline and serial pain, dyspnea, fatigue, global health, role function, analgesic use, and return to paid or unpaid work. Adjust for prior thoracic entry, lesion burden, bilateral treatment, systemic therapy, and baseline illness. Instrument- and population-appropriate thresholds for meaningful change should be prespecified; statistical significance alone is insufficient. Where PM-specific thresholds are unavailable, identify thresholds borrowed from other populations as provisional.

Contemporary radiotherapy PRO studies provide indirect methodological context. In SABR-5, 133 patients with baseline and at least one follow-up assessment generally had stable group trajectories, yet their worst individual scores showed clinically important deterioration (30). OligoCare reported baseline questionnaires for 1,033 of 2,041 enrolled patients and follow-up data for 450; mean global health was stable, while 23% reported deterioration at a median of 2.7 months (31). Both studies included multiple metastatic sites and cannot define recovery after pulmonary-only treatment or PM. They illustrate why patient-level change, questionnaire denominators, and reasons for missing data should accompany mean scores.

## 6 Repeat metastasectomy and the cumulative functional burden

### 6.1 Feasibility in selected patients is established more clearly than cumulative cost

Earlier repeat-PM studies describe low operative mortality in selected CRC and mixed-primary cohorts (32, 33), including a 2024 series of 68 repeat-PM patients with substantial sarcoma and CRC representation (34). Larger recent data qualify this reassurance. The Dutch national CRC audit included 1,237 patients, 127 of whom underwent repeat PM; postoperative complications were reported in 18.1% after repeat versus 11.6% after first PM (35). These are selected procedure groups, not a randomized estimate of the effect of reoperation. They nevertheless argue against assuming that each additional operation has the same burden.

Mills et al. studied 117 patients who reached at least a third metastasectomy and reported greater blood loss and more wound complications at the third procedure, with increasing risk of prolonged air leak across procedures (36). A 2026 mixed-primary series described 1,106 patients and 1,503 resections, including 150 patients in a repeated-resection subgroup (37). It adds long-term survival and hospital-use information, but not serial PROs or a functional counterfactual. Thus, contemporary literature expands feasibility evidence more than it resolves cumulative functional cost, consistent with the 2025 reoperative-PM review (38).

### 6.2 Histology and selection alter interpretation

CRC cohorts associate longer disease-free intervals and complete repeat resection with favorable survival (32, 39, 40). Sarcoma studies likewise show strong selection by recurrence pattern and fitness (41, 42). In a 2026 sarcoma cohort of 60 patients, 31 developed a second pulmonary recurrence and 16 underwent repeat PM (43). Its data-derived six-month disease-free-interval cutoff is a cohort-specific prognostic observation, not a validated eligibility rule. A 2023 renal-cell carcinoma series also reported feasibility of repeated resection (44), but cannot establish benefit in patients receiving current alternative systemic regimens. These histology-specific findings should not be averaged into a universal retreatment recommendation.

Direct longitudinal functional data remain sparse. In Motono et al.'s 41-patient repeat-lung-resection cohort, median vital capacity was 102.9% predicted before the first procedure and 87.4% before the second (45), a difference of −15.5 percentage points between medians. Only ten patients had metastatic tumors; intervening disease and treatment prevent attribution of this difference to PM alone. Survival after repeated surgery is additionally affected by time-dependent reselection (46). Function, persistent symptoms, hospital days, and treatment interruptions should be analyzed across episodes, including patients who do not undergo another operation.

### 6.3 Defining future local-treatment eligibility

Future local-treatment eligibility is proposed as a structured record at a subsequent decision point. For each modality, technical feasibility concerns target location, distribution, anticipated margins or dose constraints, and changes from prior treatment; physiological tolerability concerns functional reserve, comorbidity, and residual toxicity; oncological appropriateness concerns histology, disease tempo, distribution, and the intended benefit relative to systemic options. Informed patient preference is documented separately as acceptance, refusal, or uncertainty about an otherwise available option. None of these components is a validated composite endpoint, and no universal threshold is proposed.

Reasons for limitations should be recorded independently as treatment-related, disease-related, patient-related, or access-related. More than one reason may apply, attribution may be uncertain, and limitations may be temporary. Persistent toxicity with a documented temporal and clinical link to prior treatment differs from diffuse progression despite preserved lung function. Refusal of an available treatment is a preference decision, not loss of physiological capacity; an administrative delay is not technical ineligibility. Choosing SBRT, systemic therapy, or observation does not establish that surgery has become impossible.

Studies should distinguish eligibility assessed, treatment offered, patient choice, treatment scheduled, and treatment delivered, with reasons for discrepancies. The original decision cohort and the recurrence subgroup should both remain visible; deaths before reassessment are reported separately. The framework makes the established preservation principle measurable in its components, but whether any component predicts patient-valued benefit or responds to a specific technique remains to be tested. Figure 1 depicts these distinctions.

**Figure 1:**
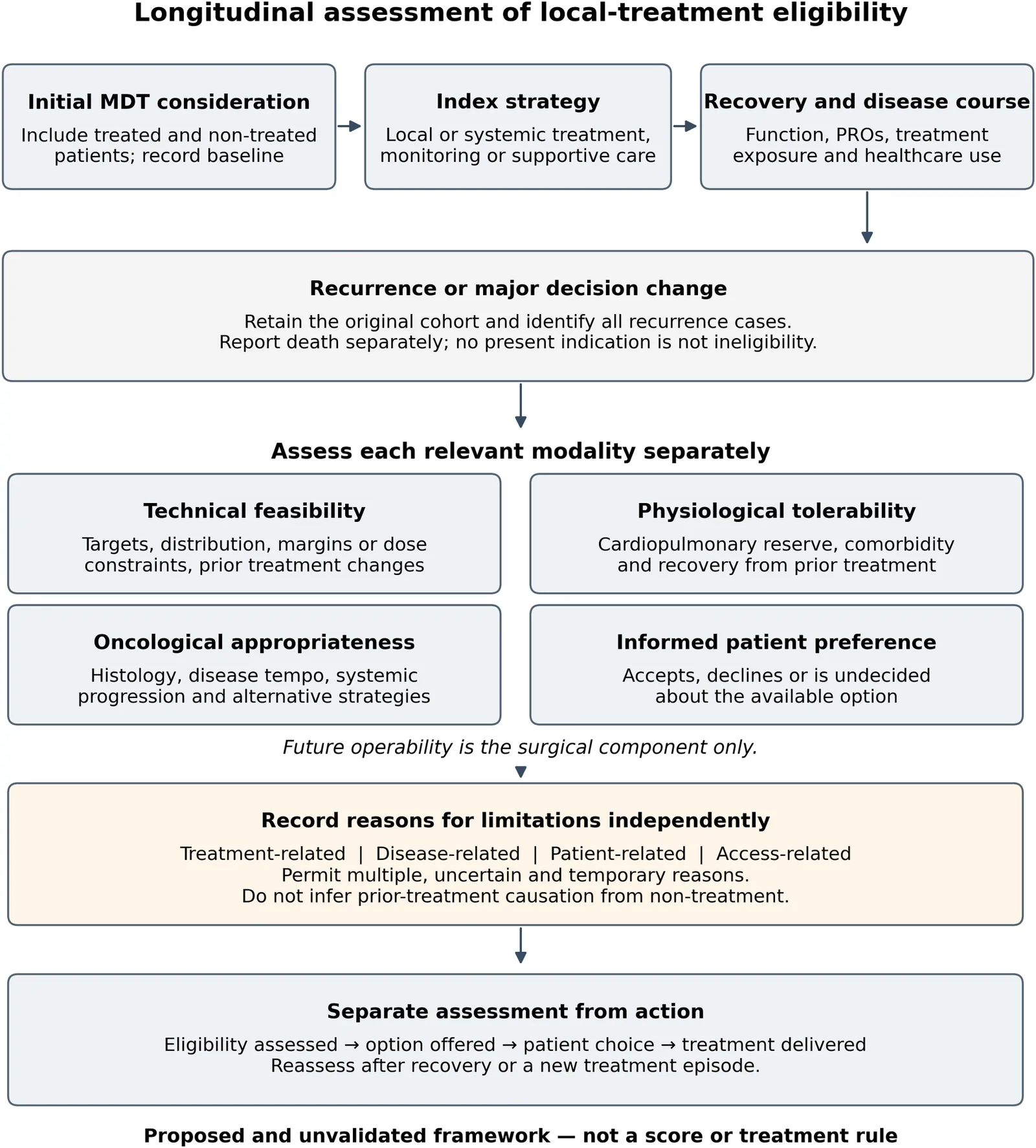
Proposed longitudinal assessment of future local-treatment eligibility. The original population considered for treatment is followed through the index intervention, recovery, and recurrence or progression. At each later decision, technical feasibility, physiological tolerability, and oncological appropriateness are assessed for each relevant modality, with informed patient preference recorded separately. Future operability is the surgical component. Reasons affecting eligibility or delivery are recorded independently as treatment-related, disease-related, patient-related, or access-related; multiple and uncertain reasons are allowed. Eligibility, the option offered, the patient's choice, and treatment delivered are distinct observations. Death is recorded separately, and absence of recurrence or an indication for treatment is not ineligibility. Pulmonary function, patient-reported health, treatment exposure, and healthcare use are measured longitudinally. This framework is proposed and unvalidated; arrows indicate assessment sequence, not causal effects or mandatory treatment pathways. MDT, multidisciplinary team; PRO, patient-reported outcome.

## 7 Non-surgical local treatment and preservation of future options

### 7.1 SBRT avoids resection but does not eliminate functional trade-offs

SBRT avoids excision and pleural entry, but these attributes do not establish superior functional preservation. Prospective pulmonary-metastasis studies support local control in selected patients (47). The SAFRON II pulmonary-function analysis randomized fractionation, not surgery versus radiotherapy: 87 of 90 randomized patients received treatment for 133 pulmonary oligometastases (48). At twelve months, modeled absolute changes from baseline were −0.17 L in FEV1 (95% CI −0.23 to −0.11) and −1.7 mL/min/mmHg in DLCO (95% CI −2.5 to −1.0). No significant between-fractionation difference was detected. The longitudinal decline cannot be wholly attributed to SBRT without an untreated comparator, but it refutes an assumption of unchanged measured function. A 2026 retrospective cohort of 55 patients reported FEV1 decline in 13 patients meeting its study-defined threshold at three months (49). Its mixed population included NSCLC, and that threshold is not a validated PM-specific meaningful-change standard.

Surgery after SABR failure remains possible in selected patients. A 2023 CRC series reported 17 patients undergoing 20 salvage resections, with grade III–IV complications after four operations and no 90-day mortality (50). Resections were more extensive than those judged potentially necessary on pre-SABR imaging. A 2026 series of 17 selected salvage cases, including 12 with CRC and two with primary lung cancer, reported two conversions to thoracotomy, one for extensive adhesions (51). These findings document technical considerations and feasibility among patients reaching surgery. Neither series provides the denominator of all recurrences eligible for salvage or separates the effect of prior irradiation from recurrent tumor growth and other causes of surgical complexity. Later options require reassessment rather than an assumed benefit or penalty from the initial modality.

### 7.2 Comparisons between SBRT and surgery remain confounded

Earlier surgery–SBRT comparisons were exploratory and subject to baseline differences (52, 53). Contemporary CRC evidence remains inconsistent in outcome definitions and allocation. In van Dorp et al.'s 2023 two-center cohort of 100 patients, local recurrence per lesion was 7.2% after surgery and 31.1% after SABR, while survival comparisons were imprecise (54). Garcia-Exposito et al.'s smaller cohort did not detect significant between-group differences in lung progression-free or overall survival (55). Neither study was designed to establish equivalence.

Lee et al.'s 2025 cohort included 334 surgical and 70 SABR-treated patients with CRC; three-year lesion-level local control was 88.6% and 86.7%, respectively, without a statistically significant difference (56). SABR patients had greater disease burden and shorter follow-up; grade 3–4 pneumonitis occurred in two of 70 patients. In sarcoma, Song et al.'s first-line survival comparison involved 61 surgical and 14 SABR patients with markedly different ages (57). These findings do not support a histology-independent modality ranking. A 2026 meta-analysis of nine retrospective studies likewise found that apparent survival advantages in the overall analysis were not statistically significant in risk-adjusted subsets (58). Loss of significance is not proof of equivalence. None of these comparisons establishes which approach better preserves longitudinal function or future eligibility.

### 7.3 Thermal ablation is parenchyma-sparing but lesion-constrained

Radiofrequency, microwave, and cryoablation avoid surgical removal of a lung wedge, but require lesion-specific access and safety assessment. A multicenter radiofrequency-ablation experience included 566 patients and 1,037 metastases (59). The ECLIPSE prospective cryoablation study enrolled 40 patients with 60 tumors and included serial quality-of-life measurement and long-term local-control follow-up (60, 61). These studies support feasibility in selected patients; they do not establish superior recovery or equivalent oncological benefit compared with surgery.

The larger SOLSTICE prospective study treated 128 patients with 224 metastases over 169 cryoablation procedures (62). Pneumothorax requiring pleural catheter placement occurred after 44/169 procedures (26%), and eight grade 3 and one grade 4 complication events were reported. SF-12 general-health scores did not show statistically significant differences at one and three months. These observations combine measurable procedural burden with reassuring group-level patient-reported results; they do not prove recovery for every patient or superiority over surgery. Lesion access, achievable margins, vulnerable structures, and diagnostic requirements remain relevant. No direct comparison establishes which local modality best preserves subsequent eligibility (Table 4).

**Table 4:** Procedural attributes, observed burdens, and unresolved comparisons.

Strategy	Procedural attribute or expected mechanism	Observed burden and evidence limits	Unresolved longitudinal question
Surgery	Removes tissue and provides histology and margins; limited resection intends to conserve lung	Function/PRO impairment documented in selected cohorts; later-resection burden requires direct measurement (9, 10, 14–18, 35, 36)	Does a specific technique preserve patient-valued function and later eligibility after adjustment for disease burden?
SBRT or SABR	Avoids excision and pleural entry; these are procedural attributes, not proof of superior recovery	Measured PFT declines; pneumonitis in comparative cohorts; technical complexity in selected salvage cases (48–51, 56)	How do dose, target location and cumulative treatment affect symptoms and later modality-specific options?
Thermal ablation	Percutaneous treatment without surgical wedge removal; tissue sampling may be possible	Local-control and QoL cohorts; procedure-level pneumothorax and drainage burden documented (59–62)	Is predicted parenchymal preservation reflected in serial function and PROs versus eligible comparators?
Systemic treatment or active monitoring	Avoids an immediate local procedure; intent and appropriateness depend on biology and context	PulMiCC offers CRC-specific randomized context, not proof that monitoring is preferable in all cases (4–7)	How do symptoms, treatment exposure and later local eligibility compare within defined histologies?

Expected mechanisms must be distinguished from measured clinical benefits. No strategy has been shown here to preserve future local-treatment eligibility better than the others. Comparisons require patients eligible for the competing options, histology-specific interpretation, and common assessment windows.

## 8 Treatment sequencing and healthcare burden

### 8.1 Local treatment should be evaluated within the systemic-treatment pathway

Local treatment must be assessed within the systemic-treatment pathway. Complications may interrupt a planned regimen, while durable local control may sometimes permit a treatment interval. Neither consequence can be inferred from R0 status. PulMiCC did not demonstrate a clear reduction in chemotherapy exposure with repeated local intervention (7). Systemic treatment alone and active monitoring are legitimate comparator strategies in defined clinical settings, not merely failures to deliver local treatment.

Record the previous and planned regimen, treatment intent, intended and actual resumption dates, dose changes, reasons for interruption, and intervening progression. Distinguish time to resumption of indicated therapy from time before a new line is required. In CRC, systemic sensitivity and molecular context influence sequencing; in sarcoma, subtype and disease tempo matter; renal-cell carcinoma and melanoma require assessment within their own systemic-treatment pathways. PulMiCC estimates cannot be transferred directly to those histologies.

### 8.2 Evidence from broader oligometastatic trials cannot be transferred uncritically

Mixed-primary and mixed-organ trials such as SABR-COMET support investigation of metastasis-directed treatment but do not establish the best modality for a particular pulmonary histology (63). Treatment-related toxicity, including fatal events reported in that trial, also matters when extrapolating a strategy of comprehensive local treatment. Such evidence is contextual, not a substitute for PM-specific comparative functional data.

### 8.3 Symptoms and resource use

The EORTC QLQ-C30 can capture cancer-related function and symptoms (64), supplemented when appropriate by selected items from the Patient-Reported Outcomes version of the Common Terminology Criteria for Adverse Events (PRO-CTCAE) (65, 66). Use a fresh baseline before each local-treatment episode rather than carrying an earlier preoperative value across chemotherapy, progression, or infection. The candidate windows are baseline within four weeks before treatment, approximately one month (2–6 weeks), three months, and twelve months, with event-triggered assessments at recurrence or major treatment changes, as in Table 3.

Resource use includes hospital and intensive-care days, readmissions, rehabilitation, toxicity management, travel, caregiver time, and loss of paid or unpaid work. Days alive and out of hospital at 30 and 90 days is a candidate integrative outcome supported by validation in broader surgical populations, not specifically validated for PM (67). Report causes of hospitalization because progression and treatment toxicity may produce the same numerical result.

Longitudinal economic evaluation also requires utilities, a stated perspective, currency and price year, time horizon, and uncertainty analysis. PulMiCC illustrates the feasibility of collecting health utilities (6), but procedure costs alone do not establish cost-effectiveness. Evidence directly linking cumulative resource use, PROs, and later eligibility after PM remains limited.

## 9 A candidate longitudinal outcome framework

Table 3 proposes candidate domains and illustrative measurement windows for feasibility work. It is not an established minimum or core outcome set, and no consensus exercise involving patients or other stakeholders has been undertaken. Formal prioritization should involve patients, caregivers, relevant specialties, and methodologists, following established outcome-set development principles (68). Instrument burden, clinical relevance, and feasible timing require testing before standardization. PRO plans should specify instruments, collection mode, missing-data handling, and meaningful-change thresholds (69). Where thresholds are not validated for the instrument and population, report continuous changes with uncertainty and label provisional interpretations. The framework supplies no eligibility score, treatment cutoff, or prescriptive imaging schedule.

## 10 Research design and clinical interpretation

### 10.1 Begin with the population considered for treatment

An inception cohort should begin at multidisciplinary consideration and retain patients assigned to surgery, SBRT, ablation, systemic treatment, observation, or supportive care. Record modality-specific eligibility and reasons for decisions before outcomes are known. Randomization among patients eligible for competing strategies remains valuable, but PulMiCC demonstrates the recruitment difficulties (4). Feasibility assessment and genuine equipoise are essential rather than assumed.

When randomization is not feasible, prospective target-trial emulation and appropriately specified adjustment can improve transparency but cannot recover unmeasured eligibility or preference. Reporting should follow observational-study principles (70). A 2026 CRC cohort of 182 patients, including 76 with recurrence, modeled salvage R0 resection as time-dependent and found an association with post-recurrence survival (71). This is a useful analytical advance, but it does not remove all confounding, includes salvage at different sites, and does not validate a PM-specific eligibility endpoint.

### 10.2 Model repeated treatment and competing events

Repeat treatment should be modeled as a time-varying exposure rather than assigning patients to a “repeat surgery” group at baseline. Multi-state approaches can distinguish recovery, recurrence, reassessment, subsequent treatment, temporary or persistent modality-specific limitations, and death. Reasons for transitions should remain visible. Treatment-related functional deterioration and disease progression are different events, although they may coexist; systemic treatment is not necessarily an absorbing state or a permanent bar to later local therapy.

Account for patient-level clustering when analyzing lesions or repeated procedures. Use competing-risk methods appropriate to the estimand and sensitivity analyses for informative missingness. Report the original decision cohort as well as recurrence-conditioned estimates. The magnitude of guarantee-time and selection bias is not quantified by this review and should not be assumed constant across cohorts.

### 10.3 Histology and framework validation

Primary histology, molecular context, systemic-treatment responsiveness, disease-free interval, growth rate, and extrapulmonary control may modify both benefit and eligibility. Prespecify these factors and avoid treating mixed-primary averages as estimates for CRC, sarcoma, renal-cell carcinoma, or melanoma. Shared measurement of physiology and patient experience is reasonable; a shared treatment indication or prognostic threshold is not.

Validation should start with explicit definitions and stakeholder consensus, followed by assessment of inter-rater agreement, feasibility, missingness, and responsiveness to changes in health or disease. Links to subsequent delivery and patient-valued outcomes should be studied without treating treatment delivery as a gold standard. A documented reason is not proof of causation: attributing loss of capacity to prior treatment requires baseline information, temporal assessment, competing explanations, and an appropriate comparative design. A retained option that the patient declines may still represent preserved capacity; an available procedure without plausible benefit is not a favorable outcome.

### 10.4 Use in multidisciplinary discussion

Clinically, the framework is a discussion aid rather than a decision rule. For example, a patient with a solitary recurrence, adequate reserve, and a technically feasible resection may choose SBRT; surgical capacity and patient choice should then be recorded separately. Conversely, preserved lung function with diffuse systemic progression may make another operation oncologically inappropriate without indicating treatment-induced functional loss. Document the treatment objective, anticipated recovery, tissue requirements, systemic alternatives, patient priorities, and uncertainty, and reassess each relevant modality at recurrence.

Shared decision-making should distinguish expected procedural differences from demonstrated patient benefits. Parenchymal preservation is an intention, not a guarantee of recovery or eligibility for another operation. A structured record may improve the transparency of decisions, but its effect on care or outcomes has not been established.

## 11 Conclusions

PM should be evaluated across a recurrent, multimodality disease course. Existing studies document early functional and symptom burdens and variable recovery in selected patients. Contemporary repeat-resection and surgery–SBRT studies add evidence of feasibility, complications, and local control, but do not establish equivalent recovery, universal histology-independent benefit, or a causal effect on retained treatment capacity.

The established principles of parenchymal preservation and repeatability motivate longitudinal measurement. Future local-treatment eligibility is proposed as a modality-specific record of technical, physiological, and oncological assessments with patient preference recorded separately; future operability is reserved for surgery. The construct remains unvalidated. Candidate outcomes and time windows require stakeholder consensus and feasibility testing. Prospective, histology-aware studies should measure function and PROs, retain appropriate denominators, and distinguish treatment-related limitations from progression, competing illness, preference, and access barriers.
