# Supplementary material for: Beyond survival after pulmonary metastasectomy: functional preservation, patient-reported outcomes, and the cumulative burden of repeat resection
Source: Front Med (Lausanne). 2026 Oct 26;13:2003447. doi: 10.3389/fmed.2026.2003447 (PMC13746041; doi:10.3389/fmed.2026.2003447)
Supplement: Supplementary file 1 [file Datasheet1.pdf]

Supplementary Material S1

Updated search and evidence provenance

Manuscript 2003447 | Searches executed 8 October 2026

This supplement documents the revision search, selection priorities, and source-access limits. It supports a narrative synthesis and does not claim a systematic-review screening process. Exact database query translations and all returned PubMed identifiers are supplied in S1_PubMed_Search_Log.json; S1_Retrieved_PubMed_Records.csv provides the deduplicated bibliographic records.

S1.1 Sources and selection

Six final focused PubMed queries were executed through NCBI E-utilities. Recent surgical queries used publication dates from 1 January 2023 through 8 October 2026. The earlier surgical function/PRO query ended on 31 December 2022; non-surgical function/PRO queries covered 2000 through 8 October 2026. No language, species, or study-design filter was applied at retrieval. English-language reports were prioritized during narrative selection. Preliminary query testing is not included in the final retrieval totals.

Priority was given to primary PM function/PRO studies, contemporary repeat-resection cohorts, and direct surgery–SBRT comparisons. Pulmonary SBRT and ablation studies were selected to examine specific assumptions about function, morbidity, and later treatment. Studies with prospective or randomized measurements were especially informative, but relevant observational cohorts were retained. Reviews supplied context and bibliographic pointers. Targeted publisher and institutional-repository searches followed up important records and identified relevant mixed-organ PRO reports, including SABR-5 and OligoCare; these were used as indirect methodological context, not pulmonary-only treatment estimates.

Selection was purposive, not independently duplicated or an exhaustive record-by-record inclusion adjudication. Pediatric-only series, isolated case reports, regional-perfusion/suffusion studies without a directly applicable comparison, and primary-lung-cancer-only studies were not prioritized. Mixed primary/secondary lung populations were retained only when they directly illustrated a relevant measurement or salvage question and were explicitly identified. No PRISMA inclusion count, formal risk-of-bias rating, pooled effect, or combined patient total is claimed. The original informal search cannot be reconstructed as a systematic screening log.

S1.2 Executed queries

Q1 Recent surgical function and PROs

Records returned: 234. Executed on 8 October 2026.

(((pulmonary[Title/Abstract] OR lung[Title/Abstract]) AND metastasectom*[Title/Abstract]) OR "pulmonary metastasis"[Title/Abstract] OR "pulmonary metastases"[Title/Abstract] OR "lung metastasis"[Title/Abstract] OR "lung metastases"[Title/Abstract] OR "pulmonary oligometastases"[Title/Abstract] OR "lung oligometastases"[Title/Abstract]) AND (metastasectom*[Title/Abstract] OR resect*[Title/Abstract] OR surg*[Title/Abstract]) AND ("quality of life"[Title/Abstract] OR "patient reported"[Title/Abstract] OR "patient-reported"[Title/Abstract] OR "lung function"[Title/Abstract] OR "pulmonary function"[Title/Abstract] OR spirometr*[Title/Abstract] OR FEV1[Title/Abstract] OR DLCO[Title/Abstract] OR dyspnea[Title/Abstract] OR pain[Title/Abstract] OR recovery[Title/Abstract]) AND ("2023/01/01"[Date - Publication] : "2026/10/08"[Date - Publication])

Q2 Recent repeat metastasectomy

Records returned: 63. Executed on 8 October 2026.

(((pulmonary[Title/Abstract] OR lung[Title/Abstract]) AND metastasectom*[Title/Abstract]) OR "pulmonary metastasis"[Title/Abstract] OR "pulmonary metastases"[Title/Abstract] OR "lung metastasis"[Title/Abstract] OR "lung metastases"[Title/Abstract] OR "pulmonary oligometastases"[Title/Abstract] OR "lung oligometastases"[Title/Abstract]) AND metastasectom*[Title/Abstract] AND (repeat*[Title/Abstract] OR reoperat*[Title/Abstract] OR iterative[Title/Abstract] OR recurrent[Title/Abstract]) AND ("2023/01/01"[Date - Publication] : "2026/10/08"[Date - Publication])

Q3 Recent surgery and stereotactic radiotherapy comparisons

Records returned: 89. Executed on 8 October 2026.

(((pulmonary[Title/Abstract] OR lung[Title/Abstract]) AND metastasectom*[Title/Abstract]) OR "pulmonary metastasis"[Title/Abstract] OR "pulmonary metastases"[Title/Abstract] OR "lung metastasis"[Title/Abstract] OR "lung metastases"[Title/Abstract] OR "pulmonary oligometastases"[Title/Abstract] OR "lung oligometastases"[Title/Abstract]) AND (metastasectom*[Title/Abstract] OR resect*[Title/Abstract] OR surg*[Title/Abstract]) AND (SBRT[Title/Abstract] OR SABR[Title/Abstract] OR stereotactic[Title/Abstract]) AND ("2023/01/01"[Date - Publication] : "2026/10/08"[Date - Publication])

Q4 Ablation function and PROs

Records returned: 127. Executed on 8 October 2026.

(((pulmonary[Title/Abstract] OR lung[Title/Abstract]) AND metastasectom*[Title/Abstract]) OR "pulmonary metastasis"[Title/Abstract] OR "pulmonary metastases"[Title/Abstract] OR "lung metastasis"[Title/Abstract] OR "lung metastases"[Title/Abstract] OR "pulmonary oligometastases"[Title/Abstract] OR "lung oligometastases"[Title/Abstract]) AND (ablat*[Title/Abstract] OR cryoablat*[Title/Abstract]) AND ("quality of life"[Title/Abstract] OR "patient reported"[Title/Abstract] OR "patient-reported"[Title/Abstract] OR "lung function"[Title/Abstract] OR "pulmonary function"[Title/Abstract] OR spirometr*[Title/Abstract] OR FEV1[Title/Abstract] OR DLCO[Title/Abstract] OR dyspnea[Title/Abstract] OR pain[Title/Abstract] OR recovery[Title/Abstract]) AND ("2000/01/01"[Date - Publication] : "2026/10/08"[Date - Publication])

Q5 Earlier surgical function and PROs

Records returned: 120. Executed on 8 October 2026.

(((pulmonary[Title/Abstract] OR lung[Title/Abstract]) AND metastasectom*[Title/Abstract]) OR "pulmonary metastasis"[Title/Abstract] OR "pulmonary metastases"[Title/Abstract] OR "lung metastasis"[Title/Abstract] OR "lung metastases"[Title/Abstract] OR "pulmonary oligometastases"[Title/Abstract] OR "lung oligometastases"[Title/Abstract]) AND metastasectom*[Title/Abstract] AND ("quality of life"[Title/Abstract] OR "patient reported"[Title/Abstract] OR "patient-reported"[Title/Abstract] OR "lung function"[Title/Abstract] OR "pulmonary function"[Title/Abstract] OR spirometr*[Title/Abstract] OR FEV1[Title/Abstract] OR DLCO[Title/Abstract] OR dyspnea[Title/Abstract] OR pain[Title/Abstract] OR recovery[Title/Abstract]) AND ("1900/01/01"[Date - Publication] : "2022/12/31"[Date - Publication])

Q6 Pulmonary SBRT function and PROs

Records returned: 95. Executed on 8 October 2026.

(((pulmonary[Title/Abstract] OR lung[Title/Abstract]) AND metastasectom*[Title/Abstract]) OR "pulmonary metastasis"[Title/Abstract] OR "pulmonary metastases"[Title/Abstract] OR "lung metastasis"[Title/Abstract] OR "lung metastases"[Title/Abstract] OR "pulmonary oligometastases"[Title/Abstract] OR "lung oligometastases"[Title/Abstract]) AND (SBRT[Title/Abstract] OR SABR[Title/Abstract] OR stereotactic[Title/Abstract]) AND ("quality of life"[Title/Abstract] OR "patient reported"[Title/Abstract] OR "patient-reported"[Title/Abstract] OR "lung function"[Title/Abstract] OR "pulmonary function"[Title/Abstract] OR spirometr*[Title/Abstract] OR FEV1[Title/Abstract] OR DLCO[Title/Abstract] OR dyspnea[Title/Abstract] OR pain[Title/Abstract] OR recovery[Title/Abstract]) AND ("2000/01/01"[Date - Publication] : "2026/10/08"[Date - Publication])

The six queries returned 728 records before cross-query deduplication and 617 unique PubMed identifiers after exact-PMID deduplication. PubMed metadata and available abstracts were retrieved for all 617 records. These

counts describe retrieval, not a denominator of eligible or included studies. Supplementary web/bibliographic follow-up is separate from these counts. A study could inform the review without appearing in one of the six targeted queries.

S1.3 Source access and extraction limits

Detailed full-text consultation was used where accessible. When full text was unavailable, interpretation was restricted to the published abstract and bibliographic record; absent follow-up, numerical units, or eligibility denominators were not inferred. An abstract-only source can support a clearly stated abstract result but cannot support unreported methodological detail. Source conclusions such as “complete recovery” or “equivalence” were not adopted automatically. Table 2 and the narrative distinguish group averages, individual changes, and procedure-level from patient-level denominators.

The matrix below covers every Table 2 entry and other references added during revision. “Full text” refers to consultation of the article in addition to its bibliographic record. Other retained foundational references were checked bibliographically; this does not imply that every original reference was newly assessed in full text. Access was not upgraded when a link led only to a publisher preview, access restriction, or abstract.

Reference and source	Role in the revised synthesis	Access used
[6] Brew-Graves C 2021<br>PMID 33002305	Primary functional/PRO or longitudinal treatment evidence	Full text and PubMed record
[9] Welter S 2013<br>PMID 23374444	Primary functional/PRO or longitudinal treatment evidence	Published abstract and bibliographic record
[10] Balduyck B 2012<br>PMID 22982657	Primary functional/PRO or longitudinal treatment evidence	Published abstract and bibliographic record
[11] Duarte D'Ambrosio P 2025<br>PMID 40246408	Contextual PM-specific PROM review	Published abstract and bibliographic record
[14] Petrella F 2009<br>PMID 19233682	Primary functional/PRO or longitudinal treatment evidence	Full text and PubMed record
[15] Welter S 2012<br>PMID 22798338	Prospective PM function and missingness	Full text and PubMed record
[16] Welter S 2016<br>PMID 25068776	Primary functional/PRO or longitudinal treatment evidence	Published abstract and bibliographic record
[17] Hassan M 2021<br>PMID 34141719	Primary functional/PRO or longitudinal treatment evidence	Full text and PubMed record
[18] Baum D 2024<br>PMID 38730714	Primary functional/PRO or longitudinal treatment evidence	Full text and PubMed record
[30] Cruz-Lim EM 2024<br>PMID 38087705	Indirect mixed-organ individual PRO trajectories	Published abstract and bibliographic record
[31] Greto D 2026<br>PMID 42314835	Indirect mixed-organ PRO and missingness example	Published abstract and bibliographic record
[35] van Dorp M 2023<br>PMID 37146204	Repeat-PM complications in CRC	Full text and PubMed record

Reference and source	Role in the revised synthesis	Access used
[36] Mills AC 2023<br>PMID 35926641	Third and later PM procedures	Published abstract and bibliographic record
[37] Ferrari M 2026<br>PMID 41670857	Long-term repeat-PM cohort	Full text and PubMed record
[38] Bribriesco AC 2025<br>PMID 40246411	Contextual reoperative-PM review	Published abstract and bibliographic record
[43] Kakibuchi D 2026<br>PMID 41575705	Sarcoma recurrence and repeat selection	Published abstract and bibliographic record
[44] Hassan M 2023<br>PMID 35987192	Renal-cell repeat-PM cohort	Published abstract and bibliographic record
[48] Bucknell NW 2024<br>PMID 37871885	Pulmonary SBRT functional trajectory	Published abstract and bibliographic record
[49] Aksoy RA 2026<br>PMID 41910296	Contemporary short-term pulmonary SBRT function	Published abstract and bibliographic record
[50] van Dorp M 2023<br>PMID 37477423	Salvage surgery after SABR	Published abstract and bibliographic record
[51] Kim EC 2026<br>PMID 41320976	Contemporary selected salvage-surgery feasibility	Full text and PubMed record
[54] van Dorp M 2023<br>PMID 37958360	CRC surgery–SABR comparison	Full text and PubMed record
[55] Garcia-Exposito N 2023<br>PMID 36831537	CRC surgery–SBRT comparison	Published abstract and bibliographic record
[56] Lee BM 2025<br>PMID 39930854	CRC local control and toxicity comparison	Full text and PubMed record
[57] Song Y 2025 (online 2024)<br>PMID 39680241	Sarcoma surgery–SABR comparison	Published abstract and bibliographic record
[58] Runkel A 2026<br>PMID 42444904	Secondary comparative synthesis	Published abstract and bibliographic record
[60] de Baere T 2015<br>PMID 26230972	Primary functional/PRO or longitudinal treatment evidence	Published abstract and bibliographic record
[61] de Baère T 2021<br>PMID 34384914	Primary functional/PRO or longitudinal treatment evidence	Published abstract and bibliographic record
[62] Callstrom MR 2020<br>PMID 32151777	Cryoablation burden and patient-reported health	Full text and PubMed record
[71] Hashimoto S 2026<br>PMID 42749975	Time-dependent CRC salvage analysis	Published abstract and bibliographic record

S1.4 Interpretation safeguards

The Welter functional and QoL reports may include overlapping participants; ECLIPSE follow-up reports describe one cohort. None is counted as an independent replication simply because it is a separate publication. The 2026 comparative meta-analysis is secondary evidence and its underlying patients are not added to Table 2 sample totals. SAFRON II randomized radiotherapy fractionation, not surgery versus SBRT. Aksoy and Kim included primary lung cancer as well as pulmonary metastases. SABR-5 and OligoCare include multiple metastatic sites and are indirect context for PRO interpretation.

Numerical checks focused on the observed study population, patient/lesion/procedure unit, assessment window, measurement scale, and permissible interpretation. The Welter FEV1 values are percentage predicted and their difference is expressed in percentage points. The Baum comparison is between cohort medians, not paired individual changes. SAFRON II is reported using absolute FEV1 and DLCO units. SOLSTICE pleural drainage is 44/169 procedures, not 44/128 patients. OligoCare follow-up results are interpreted in light of questionnaire availability, not as complete-cohort recovery.

The review remains limited by a single bibliographic database, targeted supplementary searches, purposive English-language selection, incomplete full-text access, and no duplicate screening or formal certainty assessment. Neither the proposed eligibility record nor the candidate outcomes or time windows underwent consensus development or validation. Their role is to guide future measurement and transparent reporting, not to direct treatment independently.

S1.5 Bibliographic details of revision additions

[11] Duarte D'Ambrosio P, Beushausen C, Pompili C. Impact of Metastasectomy on Patient-Reported Outcomes and Health-Related Quality of Life. Thorac Surg Clin. 2025;35(2):189-200. doi: 10.1016/j.thorsurg.2024.11.001

[15] Welter S, Cheufou D, Ketscher C, Darwiche K, Maletzki F, Stamatis G. Risk factors for impaired lung function after pulmonary metastasectomy: a prospective observational study of 117 cases. Eur J Cardiothorac Surg. 2012;42(2):e22-7. doi: 10.1093/ejcts/ezs293

[30] Cruz-Lim EM, Mou B, Baker S, Arbour G, Stefanyk K, Jiang W, et al. Prospective Longitudinal Assessment of Quality of Life After Stereotactic Ablative Radiotherapy for Oligometastases: Analysis of the Population-based SABR-5 Phase II Trial. Clin Oncol (R Coll Radiol). 2024;36(3):148-156. doi: 10.1016/j.clon.2023.11.041

[31] Greto D, Van Hemelrijck M, Oppong F, Lievens Y, Ratosa I, Jereczek-Fossa B, et al. Short-term quality of life after metastases-directed SBRT: Results of the ESTRO & EORTC OligoCare cohort. Radiother Oncol. 2026;222:111645. doi: 10.1016/j.radonc.2026.111645

[35] van Dorp M, Wolfhagen N, Torensma B, Dickhoff C, Kazemier G, Heineman DJ, et al. Pulmonary metastasectomy and repeat metastasectomy for colorectal pulmonary metastases: outcomes from the Dutch Lung Cancer Audit for Surgery. BJS Open. 2023;7(3):zrad009. doi: 10.1093/bjsopen/zrad009

[36] Mills AC, Hofstetter WL, Mehran RJ, Rajaram R, Rice DC, Sepesi B, et al. Repeated Pulmonary Metastasectomy: Third Operations and Beyond. Ann Thorac Surg. 2023;115(3):679-685. doi: 10.1016/j.athoracsur.2022.07.025

[37] Ferrari M, Valsecchi C, Leuzzi G, Rolli L, Sabia F, Pardolesi A, et al. Long-Term Results of Multiple Pulmonary Metastasectomies. Ann Surg Oncol. 2026;33(5):4313-4323. doi: 10.1245/s10434-025-18992-1

[38] Bribriesco AC, Bansal P, Subramanian MP, Bograd AJ. Reoperative Pulmonary Metastasectomy: Outcomes and Indications. Thorac Surg Clin. 2025;35(2):215-222. doi: 10.1016/j.thorsurg.2025.02.001

[43] Kakibuchi D, Okada S, Furuya T, Shimomura M, Nakazono C, Teramukai S, et al. Pulmonary metastasectomy from bone and soft tissue sarcomas: defining surgical indications at the time of second pulmonary recurrence. Int J Clin Oncol. 2026;31(3):504-515. doi: 10.1007/s10147-026-02965-9

[44] Hassan M, Ehle B, Le UT, Titze L, Passlick B, Grapatsas K. Outcome of Repeated Resection of Pulmonary Metastases for Renal Cell Cancer. Thorac Cardiovasc Surg. 2023;71(2):130-137. doi: 10.1055/s-0042-1755555

[48] Bucknell NW, Hardcastle N, Bressel M, Moore A, Montgomery R, Murnane A, et al. Impact on Pulmonary Function in a Randomized Trial of Single-Fraction and Multifraction Stereotactic Body Radiation Therapy for Pulmonary

Oligometastatic Disease: An Analysis of TROG 13.01 (SAFRON II). Int J Radiat Oncol Biol Phys. 2024;118(4):944-951. doi: 10.1016/j.ijrobp.2023.09.052

[49] Aksoy RA, Koca T, Çıtak S, Dirol H, Korcum AF. Early changes in pulmonary function after stereotactic body radiotherapy for lung oligometastases. J Cancer Res Ther. 2026;22(1):120-127. doi: 10.4103/jcrt.jcrt_2018_25

[50] van Dorp M, Ünal S, Gooijer S, Dickhoff C, van den Broek FJC, Kazemier G, et al. Salvage surgery for local recurrences after stereotactic ablative radiotherapy of colorectal pulmonary metastases. J Surg Oncol. 2023;128(7):1114-1120. doi: 10.1002/jso.27404

[51] Kim EC, Park S, Hong EJ, Na KJ, Park IK, Kang CH, et al. Salvage Resection for Local Failure after Stereotactic Ablative Radiotherapy for Pulmonary Malignancies: A Retrospective, Single-Center Study. J Chest Surg. 2026;59(3):109-114. doi: 10.5090/jcs.25.107

[54] van Dorp M, Trimbos C, Schreurs WH, Dickhoff C, Heineman DJ, Torensma B, et al. Colorectal Pulmonary Metastases: Pulmonary Metastasectomy or Stereotactic Radiotherapy? Cancers (Basel). 2023;15(21):5186. doi: 10.3390/cancers15215186

[55] Garcia-Exposito N, Ramos R, Navarro-Perez V, Molina K, Arnaiz MD, Padrones S, et al. Stereotactic Body Radiotherapy versus Surgery for Lung Metastases from Colorectal Cancer: Single-Institution Results. Cancers (Basel). 2023;15(4):1195. doi: 10.3390/cancers15041195

[56] Lee BM, Kim HE, Yang YH, Yang SY, Kim HS, Choi SH, et al. Stereotactic Ablative Radiotherapy versus Surgery in Patients with Pulmonary Metastases from Colorectal Cancer. Cancer Res Treat. 2025;57(4):1135-1143. doi: 10.4143/crt.2024.1040

[57] Song Y, Kim YJ, Choi S, Yun JK, Ahn JH, Kim JE, et al. Stereotactic ablative radiotherapy for pulmonary metastasis from sarcoma: a retrospective comparison with metastasectomy. Clin Exp Metastasis. 2025;42(1):2. doi: 10.1007/s10585-024-10320-3

[58] Runkel A, Caldonazo T, Kirov H, Mayer L, Passos FS, Mukharyamov M, et al. Metastasectomy versus stereotactic body radiotherapy for lung metastases: a systematic review and meta-analysis of reconstructed time-to-event data on overall and progression-free survival. J Thorac Dis. 2026;18(6):642. doi: 10.21037/jtd-2025-1-2674

[62] Callstrom MR, Woodrum DA, Nichols FC, Palussiere J, Buy X, Suh RD, et al. Multicenter Study of Metastatic Lung Tumors Targeted by Interventional Cryoablation Evaluation (SOLSTICE). J Thorac Oncol. 2020;15(7):1200-1209. doi: 10.1016/j.jtho.2020.02.022

[71] Hashimoto S, Tominaga T, Shiraishi T, Noda K, Yamashita M, Katayama H, et al. Salvage R0 Resection on Post-Recurrence Survival After Pulmonary Metastasectomy for Colorectal Cancer: A Multicenter Retrospective Study. Ann Surg Oncol. 2026; published online 2026-09-16. doi: 10.1245/s10434-026-20566-8
